# Pharmacological Management of Severe Neuropathic Pain in a Case of Eosinophilic Meningitis Related to Angiostrongylus cantonensis

**DOI:** 10.1155/2018/5038272

**Published:** 2018-10-17

**Authors:** Jennifer Busse, David Gottlieb, Krystal Ferreras, Jennifer Bain, William Schechter

**Affiliations:** ^1^Anesthesiology, Morgan Stanley Children's Hospital of New York-Presbyterian at Columbia University Irving Medical Center, USA; ^2^Neurology, Morgan Stanley Children's Hospital at Columbia University Irving Medical Center, USA; ^3^Anesthesiology and Pediatrics, Morgan Stanley Children's Hospital at Columbia University Irving Medical Center, New York, NY, USA

## Abstract

Angiostrongylus cantonensis, the rat lungworm, is the most common infectious cause of eosinophilic meningitis and can be fatal. The parasite can be found throughout Southeast Asia and Pacific Islands and the global distribution is expanding. We present the case of a fourteen-year-old female who had previously traveled to Hawaii and developed severe neuropathic pain related to A. cantonensis infection refractory to gabapentin and pregabalin monotherapy, who was eventually managed with an ultralow dose ketamine infusion, methadone, and serotonin-norepinephrine reuptake inhibitor.

## 1. Introduction

Neuropathic pain related to Angiostrongylus cantonensis (A. cantonensis) has not been well described but may be severe, debilitating, and difficult to treat. A. cantonensis, the rat lungworm, can be found throughout Southeast Asia and the Pacific Islands, but its prevalence has been spreading globally and cases have been identified originating in the southwestern United States [1, 2]. Infection is caused by eating raw or uncooked foods containing the infective stage larvae of A. cantonensis. A. cantonensis is the most common cause of eosinophilic meningitis (EM) [1–4]. Symptomatology is not consistent among those infected, so initial diagnosis can be challenging and effective treatment of pain symptoms is difficult. The most commonly described symptoms include severe, persistent headaches caused by increased intracranial pressure and leptomeningeal inflammation [5], paresthesias [1, 4], and cranial nerve palsies [6]. Lesions associated with A. cantonensis have been reported in both cerebral hemispheres and the cerebellum with spinal nerve root involvement being the most likely primary cause of the sensory disturbance; therefore the origin of the pain is likely central and peripheral in nature [7].

Improvement in symptoms is generally seen within 3 to 6 weeks after diagnosis and they are rarely persistent after this time [1, 3]. In unusual refractory cases, patients may develop severe permanent neurological deficits [3, 6]. For mild cases, analgesics such as acetaminophen or nonsteroidal anti-inflammatory agents suffice; however, steroids are often recommended for severe refractory symptoms. Primary anthelmintic therapy is controversial because the helminth's life-cycle is limited in humans and treatment may accelerate a robust and detrimental immune response [3, 5–10].

We present a case of A. cantonensis infection in a 14-year-old female during a trip to Hawaii who developed severe ascending neuropathic pain initially involving both distal lower extremities and the mid abdominal region. It was refractory to mild analgesics, steroids, and commonly recommended doses of gabapentin and pregabalin.

### 1.1. Consent for Publication

The authors reviewed the case report with the patient and her parents. The patient gave verbal assent and the parents provided verbal permission for the authors to publish this report.

## 2. Case Description

A fourteen-year-old 48.9 kilogram (kg) female with a history of intermittent, infrequent migraines presented to our institution's emergency department with bilateral distal leg pain, severe mechanical allodynia, and truncal rash which began two weeks previously while in Hawaii after ingestion of uncooked spinach. Initial symptoms consisted of full body itching, initially without a rash, rhinorrhea, congestion, or cough. A maculopapular rash evolved to cover her entire truncal region and thighs. She then developed intense bilateral distal lower extremity pain in a stocking-like distribution from feet to knee, which became exquisitely painful to light touch and ambulation. She described the pain as “sharp” and “shooting”. She then developed spontaneous tingling and numbness in both feet and hands, as well as tremors in all four extremities. She complained of burning pain across her abdomen at dermatome T10. Pain was rated at 10/10 and constant. She additionally complained of headache, diplopia, lightheadedness, and urinary retention. Before she was admitted to the hospital her pain was managed with acetaminophen, ibuprofen, and gabapentin. After the trial of gabapentin failed to reduce pain it was discontinued and pregabalin was started while still an outpatient.

A brain MRI, with and without contrast, was normal but the total spine MRI showed slight increased signal in the right dorsal cord especially at the level of T_11_-T_12_. A lumbar puncture revealed an opening pressure of 46 and closing pressure of 15 cm H_2_O, a protein of 82, and glucose of 54 mg/dL with leukocytosis of 390 cells/*μ*L and 17% eosinophils. Cerebrospinal fluid (CSF) serology was sent. The complete blood count (CBC) was normal except for an elevated white blood cell count of 11.46. X 10^3^ cells/*μ*L. A diagnosis of eosinophilic meningitis was made. Prednisone, 20 milligrams (mg), every eight hours was started, as were around-the-clock acetaminophen, ketorolac, and topical 5% lidocaine patches. Additionally, hydroxyzine 12.5 mg was given, as needed, for pruritus to good effect. The hydroxyzine and clonazepam given for sleep were discontinued because of excessive sedation.

Despite the above interventions, the pain remained refractory and so the following day ketamine was started at 0.02 milligrams (mg) per kilogram (kg) per hour, which was increased over five hours to 0.05 mg per kg per hour. Duloxetine, 20 mg, was administered at bedtime and methadone 2.5 mg every twelve hours was also added for continued pain that night. The following morning, the patient reported reduction in her pain to a numeric pain score of 6/10. Her leg pain resolved with the exception of the dorsum of her feet bilaterally; however, the burning pain persisted at approximately the T10 dermatome. Pregabalin continued to be slowly titrated upward to its maximum dose of 100 mg every eight hours. She did have one report of a vivid dream, but no hallucinations, tachycardia, hypertension, or signs of serotonergic or noradrenergic syndrome were present. On day 5 of admission, albendazole was started as per the recommendations of the Hawaii Department of Health. She had no additional side effects to the analgesic medications and her mental status remained normal. Diplopia, headache, and urinary retention resolved within four days of hospitalization. Ketamine was weaned and the patient was discharged with duloxetine, methadone, pregabalin, and prednisone with plans to be tapered by Pediatric Neurology as an outpatient. Of note, within two weeks of discharge, pregabalin and methadone weaning was initiated with recrudescence of pain despite continued administration of prednisone. The weaning was then restarted the following week at a slower rate and was better tolerated. CSF serologies confirmed diagnosis of A. cantonensis infection.

## 3. Discussion

This case report reflects that of a teenager infected with A. cantonensis with subsequent eosinophilic meningitis causing severe headache and neuropathic pain, which is likely both central and peripheral in origin. Neuropathic pain is known to be poorly responsive to opioids [11], as well as acetaminophen and nonsteroidal anti-inflammatory, but may often be effectively treated with anticonvulsants, such as the gabapentin and pregabalin, with minimal risk. In this case, these usual medications were not effective for the patient's pain control; however the addition of ketamine was effective.

The mainstay of most treatments for neuropathic pain includes utilization of gabapentin and pregabalin, which decrease cellular excitability through hyperpolarization [12, 13]. Other pharmacologic approaches to pain management include utilizing the antidepressant class such as amitriptyline and nortriptyline or the newer amine reuptake inhibitors such as the SNRI duloxetine, which exert their effects through a multiplicity of mechanisms of action [14, 15] including descending inhibition via the nucleus tractus solitarius [16]. Methadone [17], lidocaine infusions [18, 19], and a variety of other anticonvulsants have also been shown to be efficacious in some patients with severe neuropathic pain syndromes.

Ketamine has also been reported to be effective in the treatment of acute neuropathic pain [20]. Its putative role as an analgesic is as an N-methyl-D-aspartate (NMDA) receptor antagonist to inhibit wind-up by inducing synthesis and release of nitric oxide as well as indirectly modulating mu opioid receptor signaling and myriad other mechanisms [21, 22]. Doses of 0.2mg/kg/h to 0.5mg/kg/h of IV ketamine given for short periods have been shown to be safe in pediatric patients [23]. Because of her complex neurological presentation, we used extreme caution in our dosing of ketamine and were surprised to see a rapid effect on pain at doses many times lower than that reported in the literature.

Neuropathic pain related to A. cantonensis can be severe, persistent, and difficult to treat. Neither ketamine, methadone, pregabalin nor duloxetine has ever been reported as treatment for acute neuropathic pain in eosinophilic meningitis. In this case, ketamine, methadone, and duloxetine were used as adjuvants to pregabalin, providing a clinically observable and rapid improvement in pain scores resulting in the ability of the patient to return to functionality. While prednisone and the lumbar puncture may have had an effect on her urinary retention and headache, there was no clear clinical observation that they were beneficial for her neuropathic pain.

The ketamine infusion was selected over other pharmacologic pain treatment modalities including lidocaine infusion because the safety profile was deemed better despite the historical admonition against its use in patients with increased intracranial pressure [24]. Contemporary literature on ketamine's effect on intracranial pressure is mixed and it is not clear whether this is a dose-related phenomenon, especially when given by infusion, since it has not been studied in patients receiving low dose infusions of ketamine for pain [25]. Medications such as pregabalin, duloxetine, and methadone require titration and several half-lives to take effect and so it was thought that they would not provide the immediate relief of this patient's severe pain that ketamine might provide even though the latter exerts some analgesic effect via NMDA antagonism. To minimize the need for additional titration of ketamine and prepare for outpatient treatment, methadone and duloxetine were also simultaneously initiated. Opioids such as methadone may also increase intracranial pressure by depressing ventilation, increasing PaCO_2_, and contributing to cerebral vasodilation. Therefore, a reduced dose of 0.1 mg/kg/day divided every twelve hours was chosen as adjuvant therapy. The reduction in ICP following lumbar puncture also provided a margin of safety during initiation of both ketamine and methadone.

Once pain was under control and in preparation for discharge, ketamine was stopped and methadone, pregabalin, and duloxetine were continued with plans of weaning as an outpatient. It is of note that her pain recrudesced as pregabalin, methadone, and steroids were weaned as an outpatient but improved when she returned to original doses. She has since tolerated the steroid weaning with methadone, pregabalin, and duloxetine continuing to manage her pain.

This report presents a difficult-to-manage case of neuropathic pain, likely of central and peripheral origin. Ultimately, the patient was clinically most responsive to ketamine. Certainly of concern is administration of ketamine to patients with increased intracranial pressure. Because of this, more study is required on the effects of subanesthetic dosing of ketamine in patients at risk of increased intracranial pressure. Ketamine appears to be an excellent adjuvant that works quickly for acute neuropathic pain in patients with eosinophilic meningitis but requires caution and administration only by those trained in its use. Duloxetine and carefully dosed methadone also appeared to be of significant benefit in this patient when immediate pain relief was required in the setting of pregabalin titration.

## References

[1] Re V. L., Gluckman S. J. (2003). Eosinophilic meningitis. *American Journal of Medicine*.

[2] Eamsobhana P. (2014). Eosinophilic meningitis caused by Angiostrongylus cantonensis--a neglected disease with escalating importance. *Tropical Biomedicine*.

[3] Murphy G. S., Johnson S. (2013). Clinical aspects of eosinophilic meningitis and meningoencephalitis caused by Angiostrongylus cantonensis, the rat lungworm. *Hawaii Journal of Medicine and Public Health*.

[4] Slom T. J., Cortese M. M., Gerber S. I. (2002). An outbreak of eosinophilic meningitis caused by Angiostrongylus cantonensis in travelers returning from the Caribbean. *The New England Journal of Medicine*.

[5] Chotmongkol V., Sawanyawisuth K., Thavornpitak Y. (2000). Corticosteroid treatment of eosinophilic meningitis. *Clinical Infectious Diseases*.

[6] Alto W. (2001). Human infections with Angiostrongylus cantonensis. *Pac Health Dialog*.

[7] Clouston P. D., Corbett A. J., Pryor D. S., Garrick R. (1990). Eosinophilic meningitis: Cause of a chronic pain syndrome. *Journal of Neurology, Neurosurgery & Psychiatry*.

[8] Rosen L., Loison G., Laigret J., Wallace G. D. (1967). Studies on eosinophilic meningitis. 3. Epidemiologic and clinical observations on pacific Islands and the possible etiologic role of Angiostrongylus cantonensis. *American Journal of Epidemiology*.

[9] Wang Q.-P., Lai D.-H., Zhu X.-Q., Chen X.-G., Lun Z.-R. (2008). Human angiostrongyliasis. *The Lancet Infectious Diseases*.

[10] Ramirez-Avila L., Slome S., Schuster F. L. (2009). Eosinophilic meningitis due to Angiostrongylus and Gnathostoma species. *Clinical Infectious Diseases*.

[11] Arner S., Meyerson B. A. (1988). Lack of analgesic effect of opioids on neuropathic and idiopathic forms of pain. *Pain*.

[12] Hendrich J., Van Minh A. T., Heblich F. (2008). Pharmacological disruption of calcium channel trafficking by the alpha 2 delta ligand gabapentin. *Proceedings of the National Academy of Sciences*.

[13] Taylor C. P., Gee N. S., Su T.-Z. (1998). A summary of mechanistic hypotheses of gabapentin pharmacology. *Epilepsy Research*.

[14] Fornasari D. (2017). Pharmacotherapy for Neuropathic Pain: A Review. *Pain and Therapy*.

[15] Deng Y., Luo L., Hu Y., Fang K., Liu J. (2015). Clinical practice guidelines for the management of neuropathic pain: a systematic review. *BMC Anesthesiology*.

[16] Bridgestock C., Rae C. P. (2013). Anatomy, physiology and pharmacology of pain. *Anaesthesia and Intensive Care Medicine*.

[17] Tremont-Lukats I. W., Challapalli V., McNicol E. D., Lau J., Carr D. B. (2005). Systemic administration of local anesthetics to relieve neuropathic pain: a systematic review and meta-analysis. *Anesthesia & Analgesia*.

[18] Dworkin R. H., O'Connor A. B., Backonja M. (2007). Pharmacologic management of neuropathic pain: evidence-based recommendations. *Pain*.

[19] Tremont-Lukats I. W., Hutson P. R., Backonja M.-M. (2006). A randomized, double-masked, placebo-controlled pilot trial of extended IV lidocaine infusion for relief of ongoing neuropathic pain. *The Clinical Journal of Pain*.

[20] Zeballos J. L., Lirk P., Rathmell J. P. (2018). Low-dose ketamine for acute pain medication management: A Timely Nudge toward Multimodal Analgesia. *Regional Anesthesia and Pain Medicine*.

[21] Backonja M., Arndt G., Gombar K. A., Check B., Zimmermann M. (1994). Response of chronic neuropathic pain syndromes to ketamine: a preliminary study. *PAIN*.

[22] Max M. B., Byas-Smith M. G., Gracely R. H., Bennett G. J. (1995). Intravenous infusion of the NMDA antagonist, ketamine, in chronic posttraumatic pain with allodynia: A double-blind comparison to alfentanil and placebo. *Clinical Neuropharmacology*.

[23] Bredlau A. L., Thakur R., Korones D. N., Dworkin R. H. (2013). Ketamine for pain in adults and children with cancer: a systematic review and synthesis of the literature. *Pain Medicine*.

[24] Evans J., Rosen M., Weeks R. D., Wise C. (1971). Ketamine in neurosurgical procedures. *The Lancet*.

[25] Kramer N., Lebowitz D., Walsh M., Ganti L. (2018). Rapid Sequence Intubation in Traumatic Brain-injured Adults. *Cureus*.

